# The Rarity of Depth Refugia from Coral Bleaching Heat Stress in the Western and Central Pacific Islands

**DOI:** 10.1038/s41598-019-56232-1

**Published:** 2019-12-23

**Authors:** Roberto M. Venegas, Thomas Oliver, Gang Liu, Scott F. Heron, S. Jeanette Clark, Noah Pomeroy, Charles Young, C. Mark Eakin, Russell E. Brainard

**Affiliations:** 1 Joint Institute for Marine and Atmospheric Research, University of Hawaii at Mānoa, 1000 Pope Road, Honolulu, HI 96822 USA; 2 Pacific Islands Fisheries Science Center, National Marine Fisheries Service, NOAA, 1845 Wasp Boulevard, Building 176, Honolulu, HI 96818 USA; 3 NOAA/NESDIS/STAR Coral Reef Watch, College Park, MD 20740 USA; 4 Earth System Science Interdisciplinary Center/Cooperative Institute for Climate and Satellites-Maryland, University of Maryland, 5825 University Research Court, College Park, MD 20740 USA; 5 Marine Geophysical Laboratory, Physics Department, College of Science, Technology and Engineering, James Cook University, Townsville, QLD 4811 Australia; 6 National Center for Ecological Analysis and Synthesis, University of California Santa Barbara, California, CA 93101 USA

**Keywords:** Ecosystem ecology, Ocean sciences

## Abstract

Some researchers have suggested that corals living in deeper reefs may escape heat stress experienced by shallow corals. We evaluated the potential of deep coral reef refugia from bleaching stress by leveraging a long record of satellite-derived sea surface temperature data with a temporal, spatial, and depth precision of *in situ* temperature records. We calculated an *in situ* stress metric using a depth bias-adjusted threshold for 457 coral reef sites among 49 islands in the western and central Pacific Ocean over the period 2001–2017. Analysis of 1,453 heating events found no meaningful depth refuge from heat stress down to 38 m, and no significant association between depth and subsurface heat stress. Further, the surface metric underestimated subsurface stress by an average of 39.3%, across all depths. Combining satellite and *in situ* temperature data can provide bleaching-relevant heat stress results to avoid misrepresentation of heat stress exposure at shallow reefs.

## Introduction

Mass coral bleaching events due to anomalously warm ocean water have increased in both frequency and severity, and represent a significant threat to coral reef ecosystems worldwide^1–4^. Coral bleaching occurs when environmental stressors cause a breakdown in the relationship between corals and their algal endosymbionts^5^. Mass coral bleaching is most commonly caused by abnormally high temperatures, 1–2 °C above the usual summer maximum for several weeks and can result in widespread coral mortality^5–8^. Coral mortality, in turn, can reduce coral diversity and habitat complexity^8,9^ and negatively impact species richness, abundance, and biomass of coral reef fishes and other associated biodiversity^8,10,11^. These attendant ecological consequences of mass coral bleaching and the corresponding declines in ecosystem services (e.g., food source, fisheries, and tourism) support the need for an accurate understanding of heat stress exposure on corals.

Currently, scientists and managers primarily use satellite-derived sea surface temperature (SST) data products to understand heat stress and predict coral bleaching due to the near real-time availability, global coverage, and ease of data access. The most extensively used metric is the Degree Heating Week^12^ developed by the U.S. National Oceanic and Atmospheric Administration’s (NOAA) Coral Reef Watch (CRW) program^3^. Despite the continued improvement and validation of SST data and satellite-derived products^6,13,14^, uncertainty has continued to exist in how well the SST data and derived metrics represent *in situ* conditions on coral reefs, especially at depth^15,16^. Sources of uncertainty include: (1) depth – satellites measure the radiance from the ocean skin layer (upper ~10 μm) to derive temperature (that is typically cooler than the surface water below due to evaporative cooling) in contrast to loggers that measure at the depth of their deployment; (2) spatial scale – satellite measurements integrate across a large area (hundreds of square meters to square kilometers) compared with the point measurement of an *in situ* temperature logger; and (3) temporal scale – the frequency of satellite temperature measurements is typically once per 3–12 hours (but up to 6 times per hour in some regions), with data products derived from these reported once per day; in contrast, *in situ* measurements are typically logged once per 5–30 minutes. As infrared sensors measure the temperature within the ocean skin layer (to ~10 μm depth), SST data represent water temperature and thus can be used to compute heat stress, at the sea surface.

Because of these uncertainties, SST data only estimate the temperature at any reef site and provide a less accurate estimate of thermal variability below the surface at a particular point than does a high-quality temperature logger. Notably, the CRW evaluation of heat stress is not based on absolute temperature but rather the temperature anomaly relative to a location-specific baseline value. The accuracy of using satellite measurements from the surface to estimate conditions at depth depends on the consistency of temperature anomaly through water depth^14^. Although comparisons between SST and *in situ* are plentiful, most studies use very near-surface temperature measurements^17–21^, with fewer studies comparing SST and *in situ* temperature at greater depths^22–25^.

Under vertically-stratified conditions, many stressors vary with depth (light attenuates and temperature generally decreases^7,26,27^) leading to the hypothesis that depth provides a potential refuge for coral reefs in future climate scenarios^28^. However, even when stratification leads to lower temperatures at depth, coral colonies at depth may be acclimated to lower temperatures or a reduced level of temperature variability than those at the surface. Bleaching thresholds have been shown to vary with depth, with higher sensitivities in deeper waters^29,30^. Further, stratification does not always exist in shallow waters and temperature anomalies may not decrease with depth due to physical forcing^22,23,28,31^. This means that heat stress does not necessarily decrease with depth.

**Figure 1:**
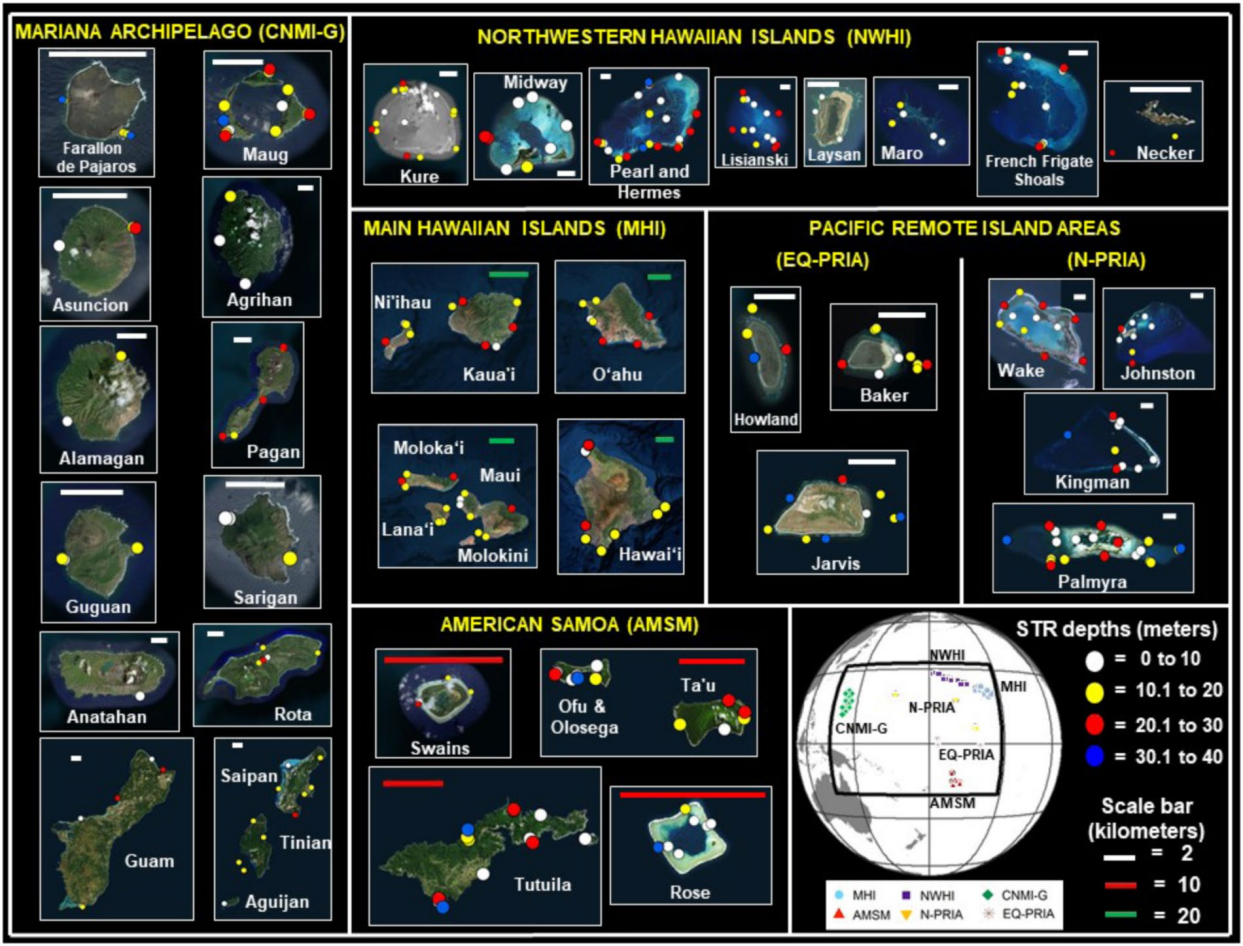
Subsurface temperature recorder (STR) study sites monitored by NOAA’s Pacific Reef Assessment and Monitoring Program (Pacific RAMP) in the six archipelagic regions: The Commonwealth of the Northern Mariana Islands and Guam (CNMI-G) with 17 islands (subsurface Santa Rosa Reef, Supply Reef, and Zealandia Bank not shown); the Northwestern Hawaiian Islands (NWHI) with nine islands (Gardner Pinnacles not shown); the Main Hawaiian Islands (MHI) with 10 islands (Lehua and Five Fathom Pinnacle not shown); American Samoa (AMSM) with six islands (South Bank not shown); the Northern Pacific Remote Island Areas (N-PRIA) with four islands; and the equatorial Pacific Remote Island Areas (EQ-PRIA) with three islands. A total of 1076 individual STR were deployed in depths ranging between 1 and 38 m at a total of 492 coral reef sites distributed among 49 islands in the six regions. Circles indicate the 457 sites with at least 365 consecutive days of data analyzed in the study. Color in circles represent STR deployment depths in meters (White = 0 to 10, Yellow = 10.1 to 20, Red = 20.1 to 30, and Blue = 30.1 to 40). Overlapping in the location of STRs (circles) is due to instrument proximity. Global map insert shows the location of the 457 sites at the six regions, and their associated names and symbols. The maps were generated by using the free version of Google Earth Pro (https://www.google.com/earth) with data from the following providers: Image Landsat/Copernicus, Image @2019 Maxar Technologies, and Image @ 2019 CNES/Airbus. The NOAA Pacific RAMP STRs location is overlapped to the individual maps.

The NOAA Pacific Reef Assessment and Monitoring Program (Pacific RAMP) has been assessing and monitoring coral reef ecosystems since 2000 across 49 islands, atolls and subsurface reefs on the western and central Pacific spanning an area of over 30 million square kilometers (Fig. 1). We divided this vast domain into six regions (Table 1): the Mariana Archipelago (comprised of the Commonwealth of the Northern Mariana Islands and Guam, CNMI-G); the Northwestern Hawaiian Islands (NWHI); the Main Hawaiian Islands (MHI); American Samoa (AMSM); the northern Pacific Remote Island Areas (N-PRIA); and the equatorial Pacific Remote Island Areas (EQ-PRIA). In this study, we utilized this large dataset of *in situ* temperature measurements from subsurface temperature recorder (STR) data collected as part of Pacific RAMP, and SST-derived products from the NOAA Coral Reef Watch program at 5-km resolution to quantify the levels of heat stress that influenced a wide range of coral reef ecosystems distributed broadly across the Pacific.

**Table 1:** Names and abbreviations of Pacific coral reef regions, islands, atolls, and subsurface reefs monitored by NOAA’s Pacific Reef Assessment and Monitoring Program (Pacific RAMP) and studied in this analysis.

Region	Islands
Commonwealth of the Northern Mariana Islands and Guam (CNMI-G, 17 islands)	Agrihan (3), Aguijan (1), Alamagan (2), Anatahan (1), Asuncion (3), Farallon de Pajaros (6), Guam (16), Guguan (3), Maug (17), Pagan (7), Rota (2), Saipan (12), Sarigan (3), Santa Rosa Reef (1), Supply Reef (1), Tinian (5), and Zealandia Bank (2)
Northwestern Hawaiian Islands (NWHI, 9 islands)	French Frigate Shoals (22), Gardner Pinnacle (1), Kure Atoll (19), Laysan (4), Lisianski (17), Maro Reef (6), Midway Atoll (9), Necker (2), and Pearl & Hermes Atoll (30)
Main Hawaiian Islands (MHI, 10 islands)	Hawai’i (10), Kaua’i (9), Lana’i (5), Lehua (3), Maui (11), Molokini (1), Molokai (7), Ni’ihau (2), O’ahu (13), and Five Fathom Pinnacle (1)
American Samoa (AMSM, 6 islands)	Ofu and Olosega (13), Rose Atoll (11), South Bank (1), Swains (7), Ta’u (9), and Tutuila (25)
Northern Pacific Remote Island Areas (N-PRIA, 4 islands)	Wake Atoll (26), Johnston Atoll (10), Kingman Reef (20), and Palmyra Atoll (40)
Equatorial Pacific Remote Island Areas (EQ-PRIA, 3 islands)	Baker (13), Howland (9), and Jarvis (16)

The number of sites at each island with at least 365 consecutive days of *in situ* data is shown in parentheses.

To better understand the potential for heat stress refugia across the western and central Pacific, we aimed to generate a site-specific metric of coral heat stress that accounts for differences across depths. To leverage the relative strengths of *in situ* and satellite temperature data, we calculated a depth-specific version of a standard measure of coral heat stress exposure, the Degree Heating Week (DHW). Satellite-derived bleaching thresholds used by CRW were adjusted for bias between the satellite records and *in situ* records at a variety of depths. Using the resulting *in situ* heat stress exposure metric, we explored the patterns of heat stress and refugia with depth across the western and central Pacific.

## Results

**Figure 2:**
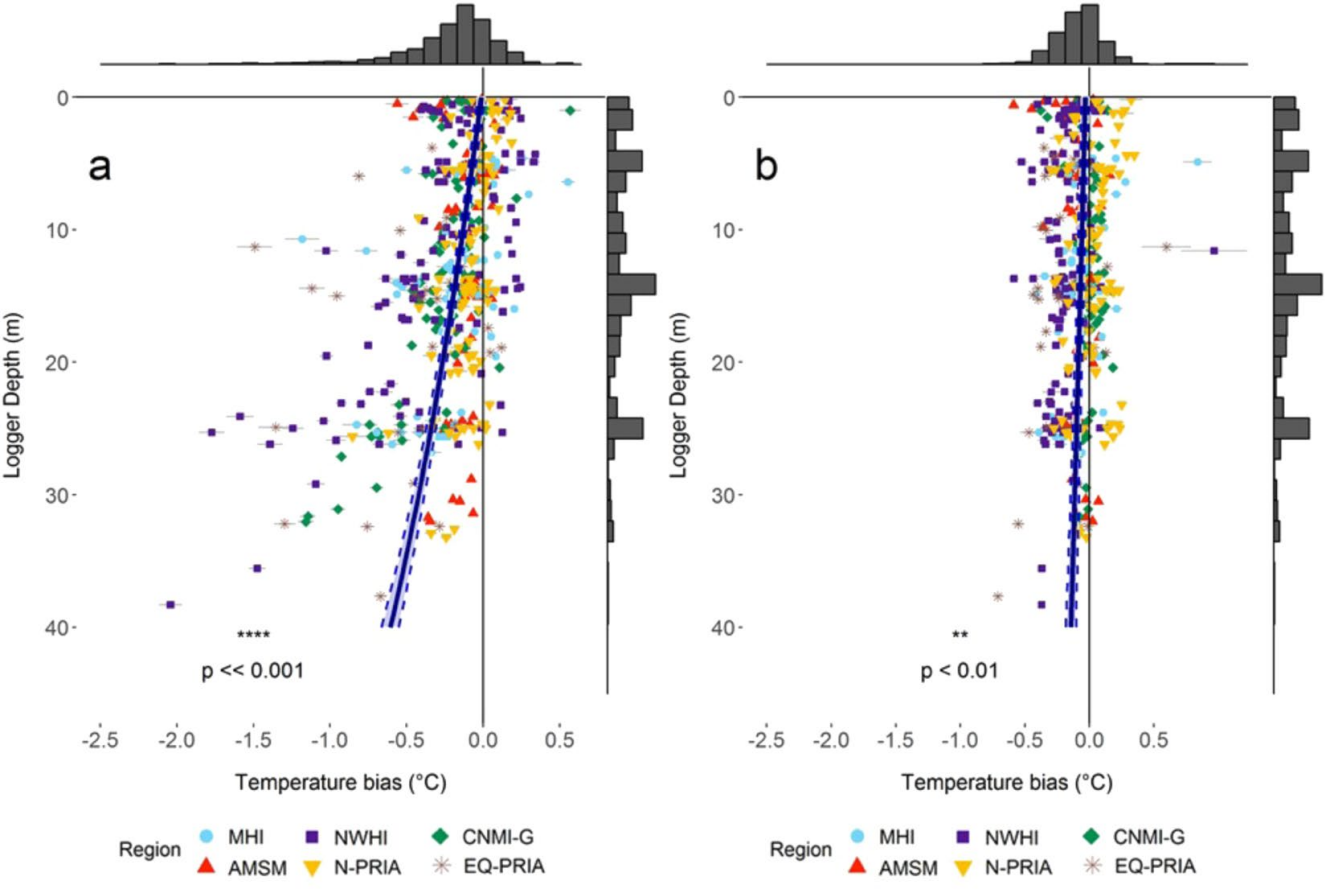
Nighttime temperature bias (*in situ* STR minus satellite SST) at depth for 457 sites from all six regions (n = 457). **(a**) Summer temperature bias during the climatologically warmest month as determined by the SST record (Fig. S8 and Table S1 shows details on statistics underlying data). (**b**) Winter temperature bias during the month that is six months offset from the summer climatologically warmest month (Fig. S9 and Table S2 shows details on statistics underlying data). In both seasons the blue line represents the linear mixed model regression and the dashed lines represent standard error of the model fit. The six regions are identified with different symbols and colors as defined in the legend. Both summer and winter temperature bias show significant association with depth (p ≪ 0.001 and p < 0.01, respectively). Histogram on top of plots refers to x-axis and represents the total count of sites associated to temperature bias bins. Histogram on right side of plots refers to y-axis and represents the total count of STRs associated to depth bins.

Long-term nighttime (local sunset to sunrise) temperature bias between SST (satellite) and STR (i.e. *in situ*) data for all sites (a site is defined as stable positions for a series of temperature loggers over time) at various depths with a full year of continuous data revealed significant temperature differences occurred with depth across the western and central Pacific (Fig. 1). These differences varied between summer (using values from the climatologically warmest month) and winter (six months offset from the climatologically warmest month) (Fig. 2).

The bias (STR minus SST) was greater in summer (Fig. 2a) than in winter (Fig. 2b). During summer the mean bias across all sites and depths was −0.21 °C (SE = 0.015), with the greatest divergence at a single site/depth of −2.04 °C (at 38 m, Fig. 2a). In contrast, bias during winter was −0.08 °C (SE = 0.008) with the greatest divergence of 0.97 °C (at 12 m, Fig. 2b). Mixed model regressions for both seasons found that bias was significantly associated with depth and its magnitude increased more rapidly with depth during summer: Negative biases (STR cooler) increased in the summer at −0.15 °C for every 10 m (Z = −12.45, p < 0.0001), compared with −0.028 °C for every 10 m (Z = −2.86, p < 0.0001) in the winter (Fig. 2a,b, respectively).

During the summer, while most of the study sites demonstrated significant cooling (increase in negative bias) with depth, the magnitude of bias varied dramatically among the six regions (Fig. S1). SST differed significantly from STR temperatures at reef depths during the summer period in all regions except AMSM. In the mixed-model framework, five of the six regions showed cooling (STR minus SST) with depth, with only AMSM showing no significant relationship between temperature bias and depth during summer (Fig. S1a). Linear mixed model for summer and winter statistics underlying data are presented in Figs. S8 and S9, respectively. The MHI and the NWHI showed the strongest depth-dependent cooling, even though at some sites small positive biases (warmer STR values) were observed between the surface and 25 m depth (Fig. S1a,b). Similar patterns of overall negative bias with occasional positive bias at some sites occurred in the N-PRIA, and the CNMI-G. The EQ-PRIA islands of Howland, Baker, and Jarvis experienced significant interannual variability associated with the El Niño Southern Oscillation (ENSO), and had some of the greatest site-based biases occurring over a range of depths and the greatest mismatch between STR and SST Maximum Monthly Mean (MMM) months (Figs. S2 and S3). Due to the interannual nature of temperature variability in the equatorial Pacific and relatively weak seasonal cycle^32^, the MMM-based method developed by CRW may not be well-suited to this region, an issue that remains to be evaluated fully.

**Figure 3:**
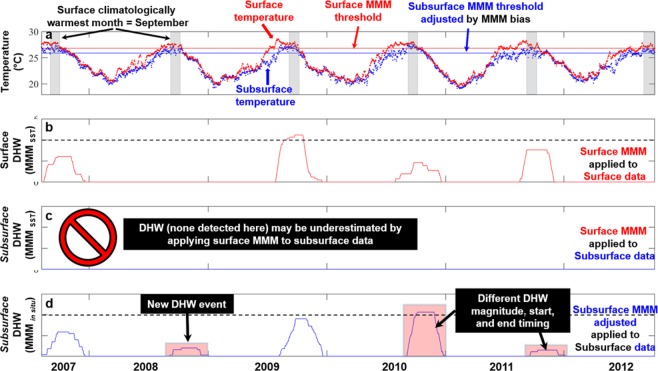
Satellite (SST) and 23 m-depth *in situ* (STR) temperature and calculated heat stress (DHW) for Pearl and Hermes Atoll, 2007–2012. (**a**) SST (red dots) and STR (blue dots) daily temperature time series with SST and STR bias-adjusted MMM thresholds (red and blue lines, respectively) for the SST climatologically warmest month (September, gray bars). (**b**) Surface DHW (MMM applied to SST surface temperature). (**c**) Subsurface unadjusted DHW (MMM applied directly to STR temperature) – the inappropriate nature of this combination is identified with the red prohibition sign). (**d**) Bias-adjusted subsurface DHW (bias-adjusted MMM applied to STR temperature). Horizontal black dashed line in (**b**,**d**) represents the threshold of DHW associated with the start of severe bleaching (i.e., DHW = 4 °C-weeks).

To illustrate our new method of calculating *in situ* coral bleaching heat stress at depth, DHW was calculated with three combinations of SST MMM thresholds and temperature data on Pearl & Hermes Atoll in the NWHI, representing one of the sites with strong stratification. We used (i) SST temperature with surface MMM; (ii) STR temperature at 23 m depth with (unadjusted) surface MMM; and (iii) STR temperature at 23 m depth with bias-adjusted MMM threshold (reflecting subsurface conditions) for the period 2007 through 2012 (Fig. 3). For the climatologically warmest month of September at the site, the bias between STR and SST at 23 m depth was −0.93 °C, defining the bias-adjusted MMM (subsurface) threshold as 0.93 °C cooler than the surface MMM (Fig. 3a).

The DHW calculated from the SST time-series and SST MMM replicates CRW’s DHW product (Fig. 3b). The result shows four heat stress events that occurred during late 2007, 2009, 2010, and 2011, each with a duration of about two months. The 2009 event accumulated more than 4 °C-weeks (a stress level for likely bleaching), whilst the remaining three events accumulated fewer than 4 °C-weeks (Fig. 3b).

The second combination applied the surface MMM directly to the STR temperature data to estimate subsurface DHW at the 23 m depth. The result highlights that assuming applicability of the surface threshold at depth can lead to potential error (Fig. 3c). This calculation resulted in no DHW events during the time period, potentially underestimating heat stress at 23 m. The data analysis on our significantly large number of study sites spreading over a large ocean basin concludes that the application of satellite MMM to *in situ* temperature data must be accompanied by a bias evaluation to avoid a likely underestimate of bleaching heat stress.

However, the subsurface DHW calculated for the 23 m depth using bias-adjusted MMM threshold reveals heat stress events at 23 m during each of the five years (Fig. 3d), one more event (in 2008) than the surface DHW reveals (Fig. 3b). Furthermore, the magnitudes of most of the subsurface heat stress events at 23 m do not match those of the corresponding surface events, although the 2009 events were similar (Fig. 3d). Notably, the magnitude of the subsurface DHW during the event in late-2010 was substantially greater (and above 4 °C-weeks; Fig. 3d) than the surface DHW (Fig. 3b). In contrast, the magnitude of the subsurface DHW during the event in late-2011 (Fig. 3d) was substantially smaller than the surface DHW (Fig. 3b).

**Figure 4:**
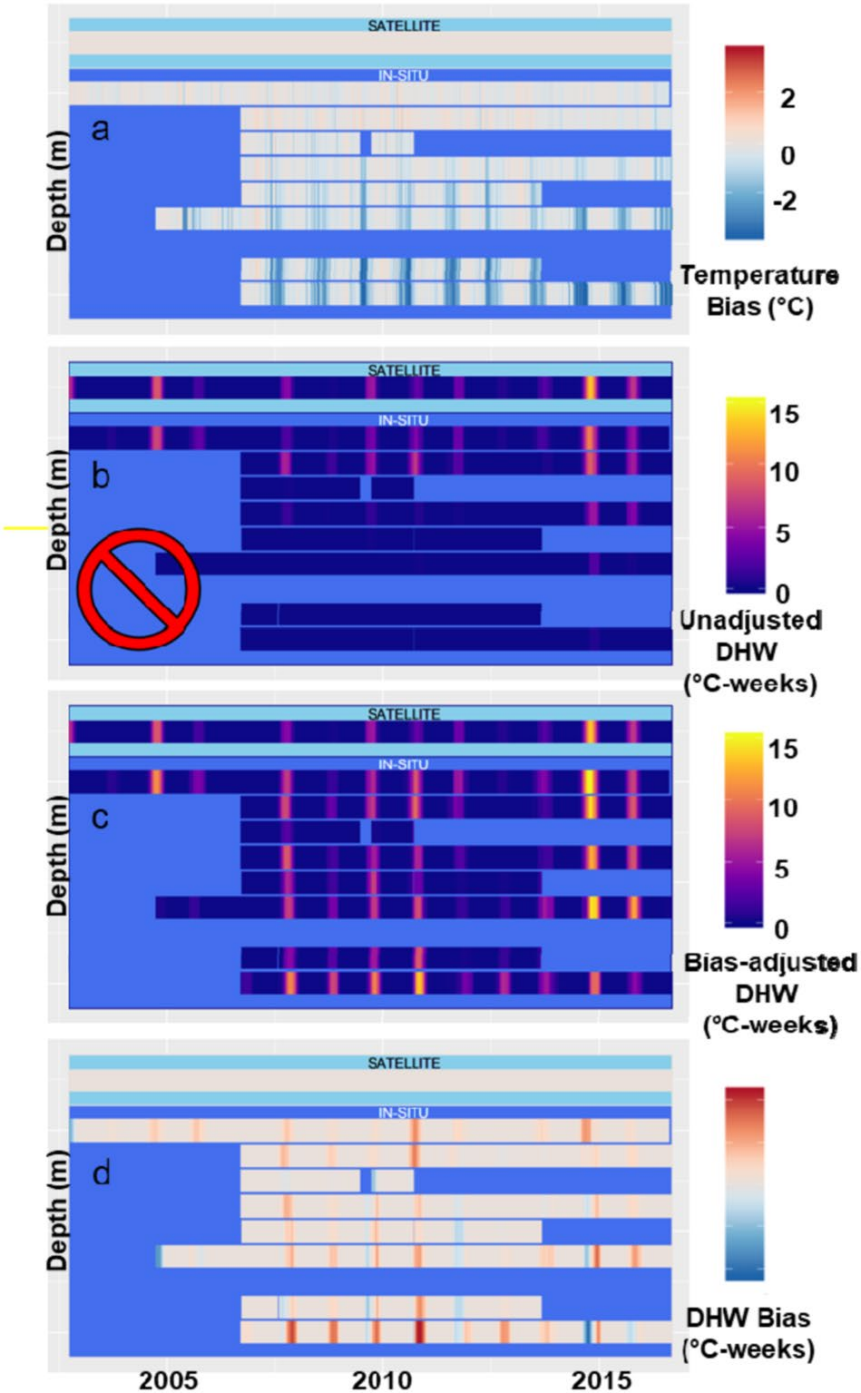
An example results of the new method of using bias-adjusted MMM to estimate DHW at depth of 38 m at Pearl & Hermes Atoll site between 2002 and 2016 (depths binned every 5 m, n = 30). (**a**) *In situ* (STR) temperature bias relative to satellite (SST) data (red = STR warmer, blue = STR cooler). (**b**) Subsurface DHW at depth using unadjusted MMM threshold (inappropriate *in situ* DHW calculation identified with red prohibition sign). (**c**) Subsurface DHW using bias-adjusted MMM threshold. (**d**) Difference between surface and bias-adjusted subsurface DHW (red indicates surface DHW is greater and blue indicates surface DHW lower than subsurface DHW).

As shown in the above example from Pearl and Hermes Atoll, adjusting the MMM threshold can have dramatic impacts on estimated levels of accumulated heat stress. To highlight these potential impacts at an island scale, Fig. 4 shows the temperature and three heat stress calculations at Pearl and Hermes Atoll between 2002 and 2016 from surface to a depth of 38 m with depths binned every 5 m. The STR temperature bias relative to SST data shows seasonally-varying temperature differences increasing in magnitude with depth (Fig. 4) and reaching −3.87 °C at 38 m (Fig. 4a). Within the top 10 m, the unadjusted subsurface DHW roughly matches surface DHW events with similar heat stress intensity up to 8 °C-weeks (Fig. 4b). Below 10 m, however, the unadjusted data artefactually suggest a refuge from stress, with few DHW positive events, and none above 4 °C-weeks, emphasizing the potential dangers of applying an unadjusted threshold to *in situ* data (Fig. 4b).

In contrast, subsurface DHW metrics using a bias-adjusted threshold (Fig. 4c) show that in most cases, heat stress events occurred from the surface to depths of 38 m with no apparent depth refuge from heat stress. Furthermore, the majority of the differences between the surface and bias-adjusted subsurface DHW (Fig. 4c,d) reveal greater heat stress at depth, especially beyond 30 m. The difference between bias-adjusted subsurface DHW and surface values (Fig. 4d) ranges from negative to positive, but positive differences appear at all depths and all years – surface DHW underestimates heat stress at depth most of the time. Notably, negative values of this difference (i.e., lower DHW at depth than in the satellite measurement) appear to be associated with a delayed onset of subsurface heat stress compared with surface values (e.g., late-2014 in the 40 m-depth bin).

**Figure 5:**
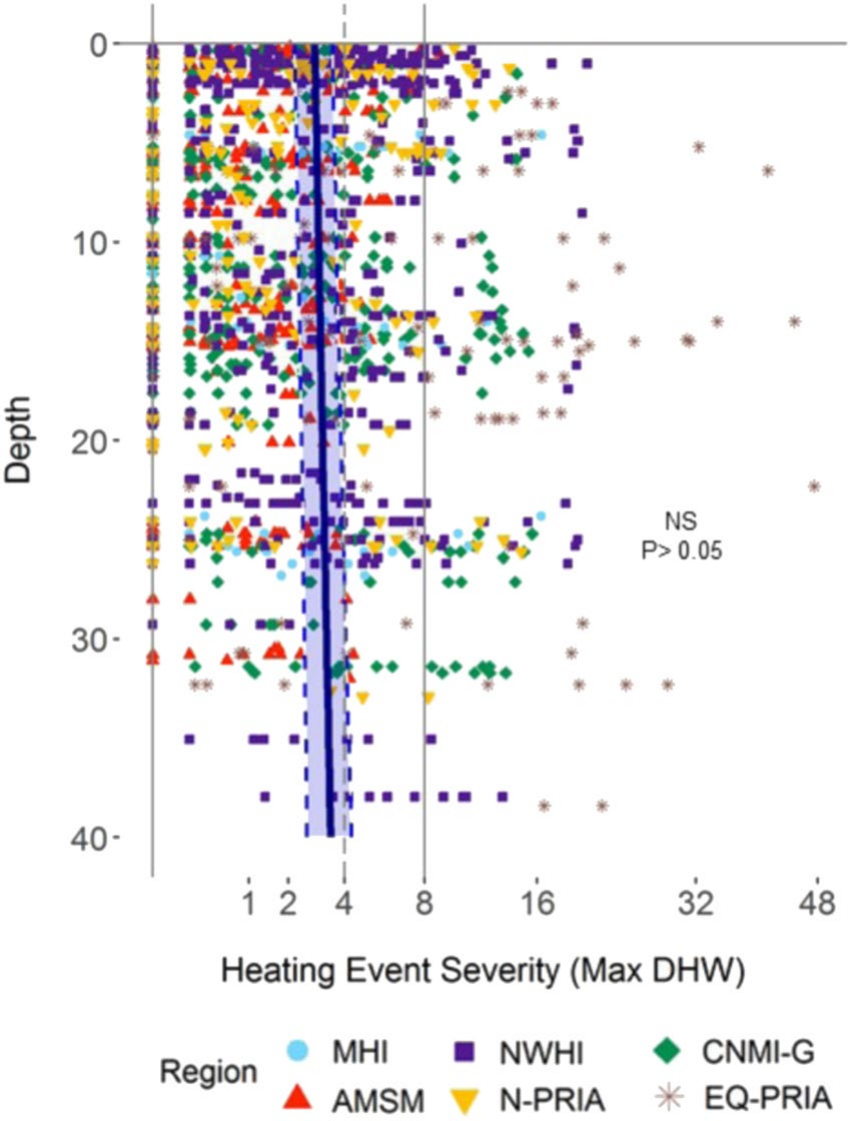
Maximum subsurface heat stress estimates for all 1,453 heating events at depth for all six regions and 457 sites across the western and central Pacific (n = 1,453). The blue line represents the linear mixed model regression fit and the blue dashed lines bound the standard error of the model fit showing no significant association between depth and observed heat stress events (NS, p > 0.05). Vertical light gray lines show DHW values reflecting likely bleaching (DHW = 4 °C-weeks, dashed) and likely widespread bleaching and significant mortality (DHW = 8 °C-weeks, solid). Fig. S10 and Table S3 shows statistical summary from linear mixed model of relationship between Maximum DHW and depth during major warming events. The six regions are identified with different symbols and colors as defined in the legend. Table S3 shows details on statistics underlying data.

Expanding our focus to sites across the central and western Pacific (Fig. 1), we assessed the relationship between depth and our *in situ* heat stress metric (bias-adjusted subsurface DHW, Fig. 5). We found no significant association between depth and subsurface heat stress considering all 1,453 recorded DHW positive heating events in our analysis (Fig. 5, Z = 1.42, p > 0.05, NS). We estimated the correlation between depth and subsurface DHW in a generalized linear mixed model (GLMM) framework, using the heating event severity as our response variable, calculated as the maximum DHW for each of the 1,453 positive DHW heating events recorded at 388 STR sites (of the total 457 sites). Controlling for site-level variation using site, island, and region as random effects, the GLMM showed that subsurface DHW calculated using bias-adjusted MMM thresholds show, collectively, no significant association between heating event severity and depth (slope: +0.05 °C-weeks for every 10 m, NS; Fig. 5). This lack of correlation between DHW and depth was apparent considering both the dataset of all heating events (N = 1,453, Z = 1.416, p = 0.157), and only those events considered severe (i.e. DHW ≥4 °C-weeks, N = 501, z = −10.24, p = 0.496). The only exception was in the CNMI-G, where we found a significant increase of DHW with depth (z = 2.626, p < 0.01). This pattern of ‘no refuge’ at depth was consistent across the six regions (Fig. S4), with no region showing a significant decrease in heat stress with depth.

**Figure 6:**
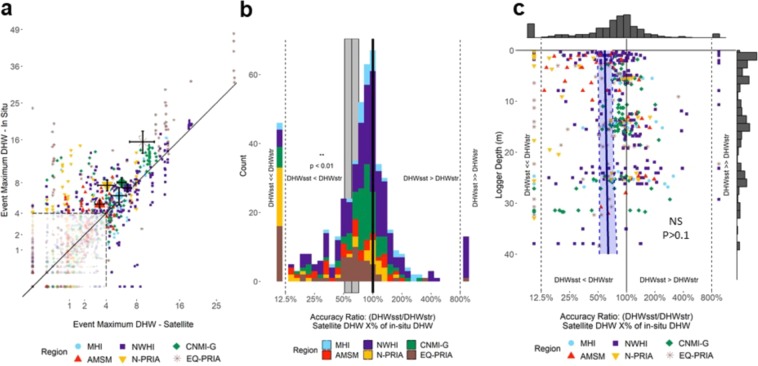
Subsurface (DHW_STR_) and surface (DHW_SST_) DHW comparison for 501 severe heat stress events (n = 501), defined as events with a maximum severity of 4 °C-weeks or greater by either metric. (**a**) Subsurface vs surface DHW maximum event severity. Region-mean DHW (only major events) is shown by a larger symbol with black whiskers indicating standard error (Fig. S11 and Table S4 shows details on statistics underlying data). Minor DHW events of <4 °C-weeks by both metrics are shown in pale colors for reference. (**b**) Distribution of ratio of surface to subsurface DHW for severe heating events (DHW_SST_/DHW_STR_), plotted on a log_2_ transformed axis (Fig. S12 and Table S5 shows details on statistics underlying data). Counts with DHW_SST_ = 0 are plotted at ratio = 10%; counts with DHW_STR_ = 0 are plotted at ratio = 1000%; and counts with both DHW values = 0 are plotted at 100%. The vertical gray bar highlights the mean mixed model estimate of ratio of surface to subsurface DHW (i.e. black line at 60.7%) with standard error of the model fit in gray (±1 standard error range: 51.4–71.7%), indicating overall underestimation by DHW_SST_ of 39.3% (**p < 0.01). (**c**) Relationship between depth and Accuracy Ratio in DHW estimates shows no significant association (NS, p > 0.05. Fig. S13 and Table S6 shows details on statistics underlying data). The six regions are identified with different symbols and/or colors as defined in the legend.

Finally, we compared surface heat stress (i.e. DHW_SST_) with the bias-adjusted subsurface DHW metric (DHW_STR_), analyzing estimates of severity from 501 severe heating events (DHW ≥4 °C-weeks or above by either metric, Fig. 6). In this comparison, the surface metrics (DHW_SST_) showed a significant tendency to underestimate subsurface heat stress (DHW_STR_) (Fig. 6a,b). Overall, the surface heat stress showed values 39.3% lower than the bias-adjusted subsurface value (Fig. 6b, mean accuracy ratio = 60.7% +/− 1 SE bounds: 51.4–71.7%, p < 0.01). The magnitude and vertical profile of this underestimation of event severity are region dependent within the spatial domain of the study: the MHI and NWHI show no significant difference between surface DHW_SST_ and subsurface DHW_STR_; in contrast, the surface value in each of the other four regions significantly underestimated subsurface heat stress (Figs. S5 and S6).

There was no consistent, significant association across the whole spatial domain of the study in the difference between surface and subsurface DHW with depth (Fig. 6c, NS, p > 0.1). However, the association of bias with depth varies dramatically, even with opposite relationships, among regions (Fig. S7). While there were no significant patterns in difference of surface and subsurface DHW with depth in the MHI, NWHI, and EQ-PRIA, both AMSM and N-PRIA showed underestimation of stress by SST primarily in the shallows (z = −2.086, p < 0.05 and z = −2.757, p < 0.01, respectively), while CNMI-G showed greater underestimation of stress by SST primarily at depth (z = −2.437, p < 0.05).

## Discussion

Due to direct and indirect effects of ocean warming, coral reefs are increasingly being affected by extreme heat stress events^14,32^, which represent one of the greatest threats to coral reefs in a changing climate^33–35^. Researchers have suggested that depth may provide a refuge for corals from bleaching^33,34,36,37^, but varying results had left this hypothesis as an area of active research^35^. The literature provides examples of both effects under heat stress: that deeper reef areas provide refuge for some coral reefs^28,31,33,36,38,39^, and also that this “deep reef refugia hypothesis” should not be assumed as a broad ecosystem-wide phenomenon^34,40^.

Across the domain of our study, depth does not provide a consistent refuge from coral bleaching heat stress down to ~35 meters. Our results indicate it is likely that many deeper coral reefs are at least as vulnerable to climate variability and change as shallower reefs^35^. After adjusting for depth-dependent thermal biases, we show coral heat stress event severity does not decline with depth across the western and central Pacific domain of our study (Fig. 5). This result is particularly striking given the apparently robust but artefactual refuges present in STR data analyzed without appropriate bias adjustment (e.g. Fig. 4b). The lack of a depth refuge from subsurface heat stress is consistent across the entire spatial domain of our dataset as well as at individual regional scale (Fig. S4). It is notable that the only significant regional scale association of bias-adjusted subsurface DHW event severity and depth was in CNMI-G, where the DHW increased with depth (Fig. S4).

Our results also show that relying on SST data alone could pose the potential risk of underestimating subsurface heat stress, as using satellite products alone tends to report levels of stress 39% lower, on average, than those estimated by our *in situ* DHW (Fig. 6). Surface estimates of DHW provide an excellent perspective of what’s occurring on broad spatial scales, but our results suggest that what’s occurring at specific points on reefs can be substantially worse than surface estimates alone would suggest. Surface heat stress substantially differed from bias-adjusted subsurface metrics in about half of the events we examined, counting both over- and under-estimation; i.e. 52.1% of events (261 of 501) had greater than 30% difference (Fig. 6b). Among these discrepancies, underestimation is arguably more cause for concern and occurred over twice as often as overestimation.

The ability of surface heat stress measurements to represent subsurface conditions varies by region, site, and sometimes even time. These variations make it difficult to simply state under which contexts surface values (DHW_SST_) will be representative of subsurface conditions (DHW_STR_). We found no consistent association between depth and the difference between surface and subsurface DHW across the domain of our analysis (Figs. 6c, S7). This would suggest that, after correcting the MMM threshold for known depth-dependent biases, the surface DHW, on average, represents subsurface conditions as well at depth as it does in the shallows. However, at the regional scale, there was substantial variation in the representation. More specifically, in some regions there was no pattern with depth (MHI, NWHI, EQ-PRIA), in some there was apparent underestimation primarily in shallower depths (AMSM, N-PRIA), and in others underestimation was primarily in deeper sites (CNMI-G; Fig. S7). In two regions, MHI and NWHI, there was no significant difference between surface and subsurface DHW across all depths, suggesting that subsurface conditions are generally well represented by surface values. Further study on these patterns is needed. Coral reefs can experience horizontal temperature gradients and temporal variability on scales that SST products cannot resolve, and in some cases this difference in scale can lead to incorrect assessment of bleaching heat stress in specific parts of reefs at specific times^3,41,42^. This study provides a method to enhance the service provided by near real-time satellite monitoring at reef sites with sufficient *in situ* temperature data records.

There are four main caveats to our conclusions. First, the adjustments we apply to bleaching thresholds *in situ* mirror those used for regional adjustment of satellite bleaching thresholds^3^, but are not based on *in situ* biological bleaching observations. The regional corrections of satellite records have performed well in modeling bleaching^3,13^, but whether this pattern is consistent across depths has yet to be shown at scale. Confirming that these *in-situ* adjustments are consistent with biological responses will be a focus of further work. Second, our method rests on the current definition of the MMM threshold by NOAA’s CRW, which, due to the fact it assigns a ‘summer’ month (i.e. the climatologically warmest month), assumes some degree of annual seasonality. Our calculations assumed that the warmest month used by CRW at the surface applies at depth as well, which is generally, but not universally true (Figs. S1 and S2), especially for deeper water depths. We observed some important deviations between SST warmest month and STR warmest month in the EQ-PRIA, where the assumption of consistent seasonality does not hold^43^ (Figs. S1 and S2). Even in the face of that variability, we believe that using the long-running SST data is a more responsible choice than using the available shorter term *in situ* data to define the warmest month. Third, given the relatively short-time series from most *in situ* datasets, we assume that the years of data used to define the bias between SST and STR time series are representative of all years. This assumption becomes more tenuous for shorter time series and under varied climatic conditions such as positive and negative phases of ENSO, as such events in the *in situ* record make it potentially less representative of the long-term climatology. Finally, although discussions of the deep refugia hypothesis often include or invoke the poorly studied mesophotic depths from 30 to 150 m, our dataset does not extend beyond 38 m, so only provides a perspective on the presence or absence of thermal refugia to that depth. However, with more distinctive thermal conditions in mesophotic depths compared with the sea surface, the proposed method may be even more applicable to deeper mesophotic temperature datasets – this remains to be tested.

The patterns we show assume that coral bleaching thresholds track a location’s long-term maximum temperatures, and as long-term temperatures tend to decrease with depth (Fig. 2a), our modeled thresholds tend to decrease with depth as well. As the thresholds we use are modeled on *in-situ* temperatures and not on realized patterns of biological bleaching response, our results may miss other important drivers of bleaching thresholds, including light, water quality, and species/colony-specific performance. Therefore, our results do not imply that there would never be respite from coral bleaching at depth, just that heat stress events above location-specific baselines do not broadly decrease with depth.

Light exposure also plays a major role in causing bleaching^27^, and is attenuated with depth even in oligotrophic coral reef waters (albeit more slowly)^44,45^. A number of studies have shown bleaching declining with depth^31,33,36,38,39^, but others have shown either increasing bleaching at depth or no relation with depth^34,35,37^. As the coral-algal holobiont can adapt to local conditions of temperature and light, the interaction of exposure to heat stress, exposure to light, the bleaching tolerance of a given coral, water quality, and past heat stress exposure (among other stressors) make bleaching responses complex to model. Our results suggest that to best model bleaching will require at least accurate models of both heat and light exposure at the coral^27^.

While our two major conclusions (i.e. that heat stress exposure does not decline reliably with depth, and satellites tend to under-estimate *in situ* heat stress) here are “bad news” for coral reef persistence, the method we apply does identify a substantial number of sites that show reduced *in situ* heat stress relative to their satellite records. Of all 257 sites with major bleaching events (DHW >4), 111 (43%) of them showed at least one event in which *in situ* stress was less than that reported in the satellite (Fig. 6). These “relative refugia” sites are in the minority in our dataset, but may be of particular interest to researchers and managers. For example, a disproportionate number of these sites (81/111; 73%) occur in the Northwest Hawaiian Islands (Fig. S6C), a region characterized by internal wave activity during stratified summer conditions. The technique we apply here can serve to identify such regions of relative refugia to aid in management.

Comprehensive geographic and temporal coverage is a universally acknowledged strength of satellite-derived SST data, at least since the beginning of the ‘satellite era’ in the early 1980s^41^. Subsurface *in situ* temperature datasets rarely match the 35-year coverage and don’t compare with the spatial repleteness that today’s best SST products can provide^41,46^. However, *in situ* time-series observations provide location-specific information, accurately representing variation across small spatial scales, sub-daily time scales, and down to any depths a temperature recorder can be deployed^25^. By combining these data types as we have done here, the long climatological reach of SST data and the location-specific accuracy of subsurface *in situ* data can be combined to provide more accurate estimates of physiologically-relevant heat stress to corals. Our analysis may help to enable identification of undetected bleaching at depth, as well as actual rare depth refuges and their characteristics. We also expect the method can provide relevant meaning for coastal managers and be applied to output from climate modelling to better understand the impact future climate change may bring to reefs.

## Materials and Methods

### Study sites and data description

The STR data were collected at coral reef study sites monitored by NOAA’s Pacific RAMP in the western and central Pacific spanning an area of 31,074,540 km^2^ (Fig. 1 and Table 1) across six regions: the Mariana Archipelago (Commonwealth of the Northern Mariana Islands and Guam, CNMI-G); the Northwestern Hawaiian Islands (NWHI); the Main Hawaiian Islands (MHI); American Samoa (AMSM); the northern Pacific Remote Island Areas (N-PRIA); and the equatorial Pacific Remote Island Areas (EQ-PRIA). The Pacific Remote Island Areas were divided into two subregions, N-PRIA and EQ-PRIA, based on the significant oceanographic differences observed between the two groups^32^.

The individual STR positions within these six regions were selected to provide baseline observations of the prevailing spatial and temporal thermal conditions necessary to understand and predict the ecological impacts of climate change on coral reef ecosystems. A total of 1076 individual STRs, with an accuracy of ±0.002 °C (SeaBird Electronics recorders SBE 39 and 56 models), were deployed in depths ranging between 1 and 38 m at a total of 492 coral reef sites distributed among 49 islands, atolls, and subsurface reefs within the six regions. The STRs were deployed at sites for multiple years started as earlier as 2002 and replaced every 2–3 years until 2017. Among these 492 sites, 457 sites (93%) with at least 365 days of continuous data were analyzed.

SST and heat stress time series data for the same time period (2002 to 2017) than STR data were extracted from NOAA Coral Reef Watch’s (CRW) daily global 5-km v3.1 coral bleaching heat stress product suite that is based on CRW’s CoralTemp v1.0^41,47^ SST dataset. SST data were extracted from the 5-km pixels that contain or were nearest to the survey sites.

### SST vs STR Bias analysis and threshold adjustment

For each of the 457 sites, we calculated the bias between coincident SST (nighttime temperature records) and STR nighttime (local sunset to sunrise) daily-averaged temperature records available within the years 2001–2017. We define an STR site as a stable position for a series of temperature loggers over time. We calculated the difference between daily, nighttime-only temperatures from STR and SST records (STR-SST) for each STR site to examine overall patterns in the daily temperature difference with depth and region (Figs. 2 and S1a,b). We evaluated the correlation between mean daily temperature bias and depth using linear mixed regression models that included region and island, as random effects. Statistical models of temperature bias were calculated using the *glmmTMB* function from the R package *glmmTMB*, using Gaussian errors with an exponential transform, and evaluated for uniformity of residuals, overdispersion, zero-inflation, and heteroscedasticity using the R package *DHARMa*^48^.

We used the temperature threshold of CRW’s daily global 5 km v3.1 product suite to determine coral bleaching heat stress at the surface. This “MMM” (maximum of the monthly mean climatologies) reflects the climatologically warmest month based on SST data from 1985–2012^3^ (Fig. 3a). Throughout our analysis, we used the warmest month as defined by the CRW’s multi-decadal SST datasets, which exactly matches the STR observed warmest month 45% of the time, is offset by one month or less 83% of the time, and is offset by two months or less 96% of the time (Figs. S1 and S2). Note: per the CRW methodology, DHW is accumulated only for SST values that are at least one degree Celsius above the MMM threshold.

To examine seasonal patterns, which varies per region, in bias between STR and SST we calculated bias across all months; during the climatologically warmest month from SST data (representing summer); and during the month that is six months offset from the climatologically warmest month (representing winter).We estimated the bias between STR and SST data at each depth during the warmest month across all years for which STR data are available, as the mean of daily differences between the STR and SST data (see Fig. 3a, dark bars). Finally, we subtracted the depth-dependent bias from the surface MMM threshold to obtain the bias-adjusted subsurface MMM threshold at each depth for each location.

### DHW calculation

The DHW, as developed by Coral Reef Watch (CRW)^3^, is a cumulative measure of the intensity and duration of coral bleaching heat stress, and is expressed in the unit °C-weeks. The DHW calculation requires two inputs – (i) daily temperature values experienced and (ii) a threshold for expected summertime temperature (MMM). DHW is calculated by subtracting the MMM threshold from the temperature time series, then summing anomalies greater than or equal to 1 °C over a rolling 12-week (84-day) window. Here, we calculated DHW using three combinations of inputs: (i) applying the SST MMM threshold to SST data, emulating the CRW product (SST DHW, Fig. 3a,b); (ii) applying the SST MMM threshold directly to STR data to generate STR DHW (Fig. 3c); and (iii) applying the STR bias-adjusted MMM threshold, based on the calculations defined in the *SST vs STR Bias Analysis and Threshold Adjustment* section, to STR data to generate the STR bias-adjusted DHW (Fig. 3d). SST and SST DHW timeseries are available for each location, while STR temperatures, STR non-bias adjusted DHW, and STR bias-adjusted DHWs are available for each depth at each location. DHW values between 4 and 8 °C-weeks are taken to indicate likely bleaching, while 8 °C-weeks and above indicate likely widespread bleaching and significant mortality^36^.

### Event analysis

We analyzed all 457 sites for the effect of adjusting the MMM threshold. Heat stress events were identified as periods during which any of our DHW estimates (SST, STR unadjusted, STR bias-adjusted) were non-zero. For each of the 1,453 events recorded, we noted the maximum DHW achieved (event max DHW) for each of the three methods. Exceptions to this occurred at the beginning or end of an STR time series, in which cases we noted maximum severity observed during the period of STR data availability.

### Heat stress with depth

We used the event-scale maximum STR bias-adjusted DHW (Fig. 3d) as the response variable for a set of GLMMs. In the GLMMs, we correlated event-scale maximum STR bias-adjusted DHW with depth, while holding the site, island, and region in which the event was recorded as random variables in the model. As these models were fit using zero-inflated negative binomial errors (i.e. a discrete distribution), the response variable was rounded to the nearest DHW integer value. All models were calculated using the *glmmTMB* function from the R package *glmmTMB*, and evaluated for uniformity of residuals, overdispersion, zero-inflation, and heteroscedasticity using the R package *DHARMa*^48^. To determine the most appropriate model, results were compared among models with: Gaussian errors using a range of transformations (square root, third root, log); models with zero-inflated negative binomial errors; and models with zero-inflated Poisson error. All Gaussian-error models showed significant overdispersion and zero-inflation, and were therefore discarded. In contrast, negative binomial and Poisson model results were similarly well-behaved with regard to overdispersion and zero-inflation, but the negative binomial model outperformed Poisson in both uniformity and goodness of fit. Therefore, negative binomial models were chosen for further analysis.

### SST vs STR bias-adjusted DHW severity

We also evaluated the ability of the surface heat stress to estimate subsurface values by examining deviations from a 1:1 correlation between the surface and bias-adjusted subsurface DHW (Fig. 6a), and the difference between these (Fig. 6b,c). As our statistic of difference, we used the “Accuracy Ratio”, i.e., the ratio of surface to subsurface maximum DHW recorded in each event (DHW_SST_/DHW_STR_), which we log_2_-transformed in both visualizations and statistics. All descriptions of results of the Accuracy Ratio, however, have been back-transformed into percentages based on the ratio. In plots, instances where DHW_STR_ was zero were treated as a large, fixed value (800%, i.e. a log_2_ ratio of +3), but in statistical test, these values were removed as they generated models with poor fits. Similarly, where DHW_SST_ was zero a small fixed value (12.5%, i.e. a log_2_ ratio of −3) was assigned for plots, but these values were excluded in the statistical test. Instances with both values equal to zero (rarely present, only as an artefact of rounding fractional DHW events) were treated as accuracy ratios of 100% or log_2_ accuracy ratios of 0, in both plots and statistics.

## Supplementary information

Supplementary Information

## Data Availability

Raw *in situ* temperature data used in this study can be downloaded from https://www.nodc.noaa.gov/access/index.html. Satellite-derived temperature data used in this study can be downloaded from CRW website (https://coralreefwatch.noaa.gov/satellite/index.php).
