# Supplementary material for: The Rarity of Depth Refugia from Coral Bleaching Heat Stress in the Western and Central Pacific Islands
Source: Sci Rep. 2019 Dec 23;9:19710. doi: 10.1038/s41598-019-56232-1 (PMC6928217; doi:10.1038/s41598-019-56232-1)
Supplement: Supplementary file 1 — Supplementary Information [file 41598_2019_56232_MOESM1_ESM.pdf]

The Rarity of Depth Refugia from Coral Bleaching Heat Stress in the Western and Central Pacific Islands

Roberto M. Venegas[1,2*], Thomas Oliver[2*], Gang Liu[3,4], Scott F. Heron[5,3], S. Jeanette Clark[6], Noah Pomeroy[1,2], Charles Young[1,2], C. Mark Eakin[3], Russell E. Brainard[2]

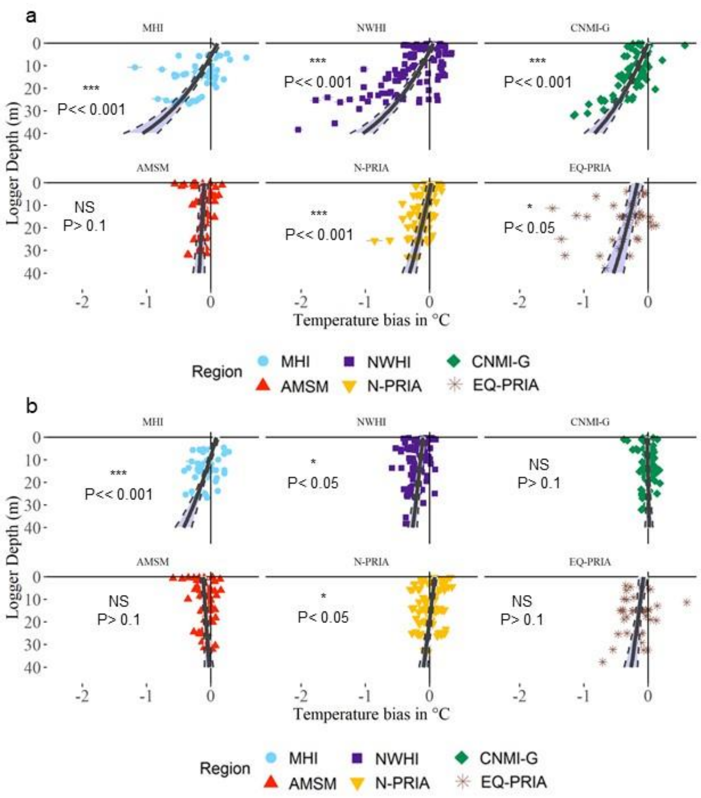

Fig. S1: Nighttime temperature bias (*in situ*, STR, minus satellite, SST) at depth for six regions across the western and central Pacific. (n=457 sites total, MHI=62, NWHI=110, CNMI-G=85, AMSM=66, N-PRIA=96, E-PRIA=38). a) Summer temperature bias during the climatologically warmest month. b) Winter temperature bias (during the month that is six months offset from the climatologically warmest month). In both seasons the gray line represents the linear mixed model regression and the dashed lines represent standard error of the model fit. Statistical significance of the association between temperature bias and depth for summer and winter in each of the six regions is shown: NS = not statistically significant with p>0.1, while *** = statistically significant with p<0.001. The six regions are identified with different symbols and colors as defined in the legend. For comparison, Figure 2 shows the basin-wide analysis.

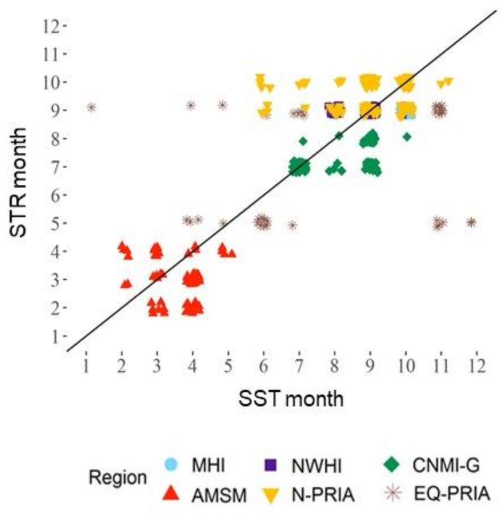

Fig. S2: Relationship of *in situ* (STR) and satellite (SST) maximum climatological month timing across the western and central Pacific, comparison of warmest month at surface and depth (n=457).

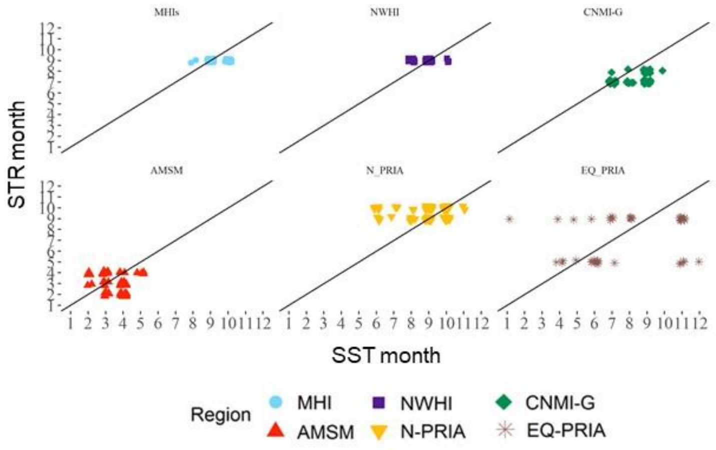

Fig. S3: Relationship of *in situ* (STR) and satellite (SST) maximum climatological month timing for six regions across the western and central Pacific, comparison of warmest month at surface and depth. (n=457 sites total, MHI=62, NWHI=110, CNMI-G=85, AMSM=66, N-PRIA=96, E-PRIA=38).

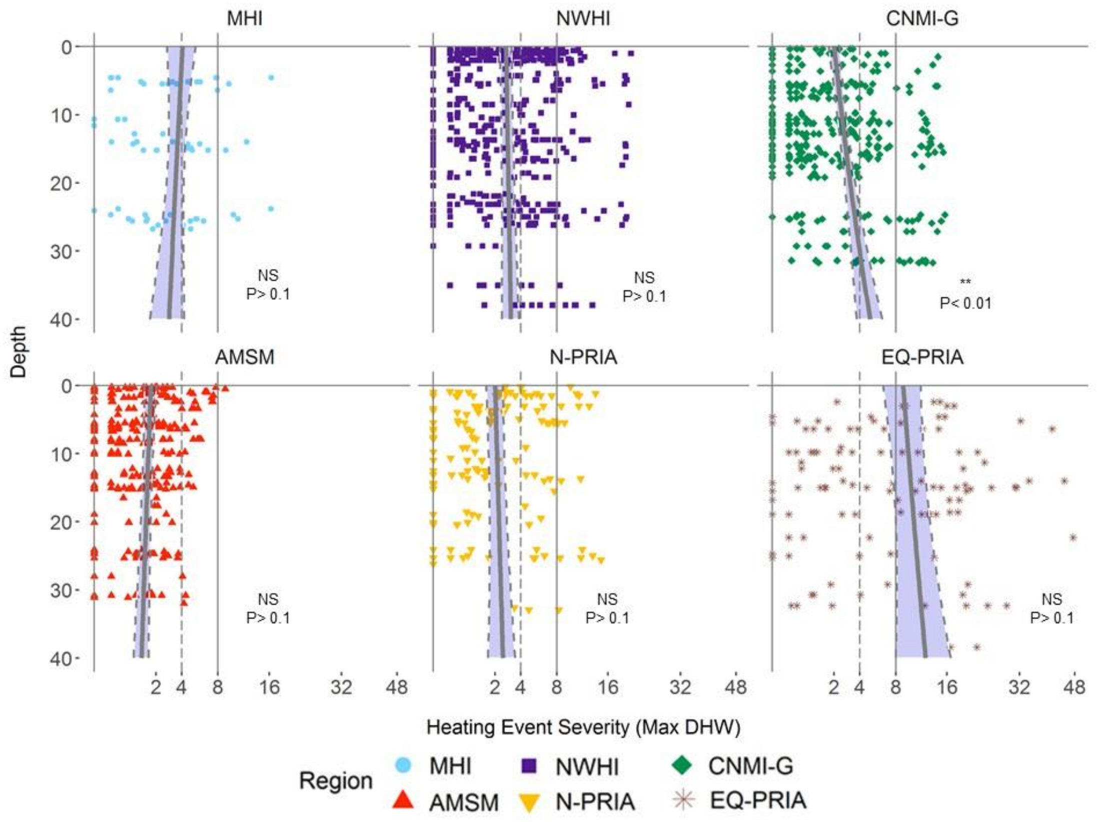

Fig. S4: Maximum bias-adjusted subsurface heat stress for six regions across the western and central Pacific. (n=1,453 events x sites total, MHI=65, NWHI=504, CNMI-G=336, AMSM=277, N-PRIA=161, E-PRIA=110). Gray solid line represents the linear mixed model regression and the gray dashed lines represent standard error of the model fit. No significant association between depth and observed heat stress was observed except for CNMI-G. Statistical significances between maximum heat stress and depth at each of the six regions are shown: NS = not statistically significant with p>0.05, and ** = statistically significant with p<0.01. Vertical light gray lines show DHW values reflecting likely bleaching (DHW = 4ºC-weeks, dashed) and likely widespread bleaching and significant mortality (DHW = 8ºC-weeks, solid). The regions are identified with different symbols and colors as defined in the legend. For comparison, Figure 5 shows the basin-wide analyses.

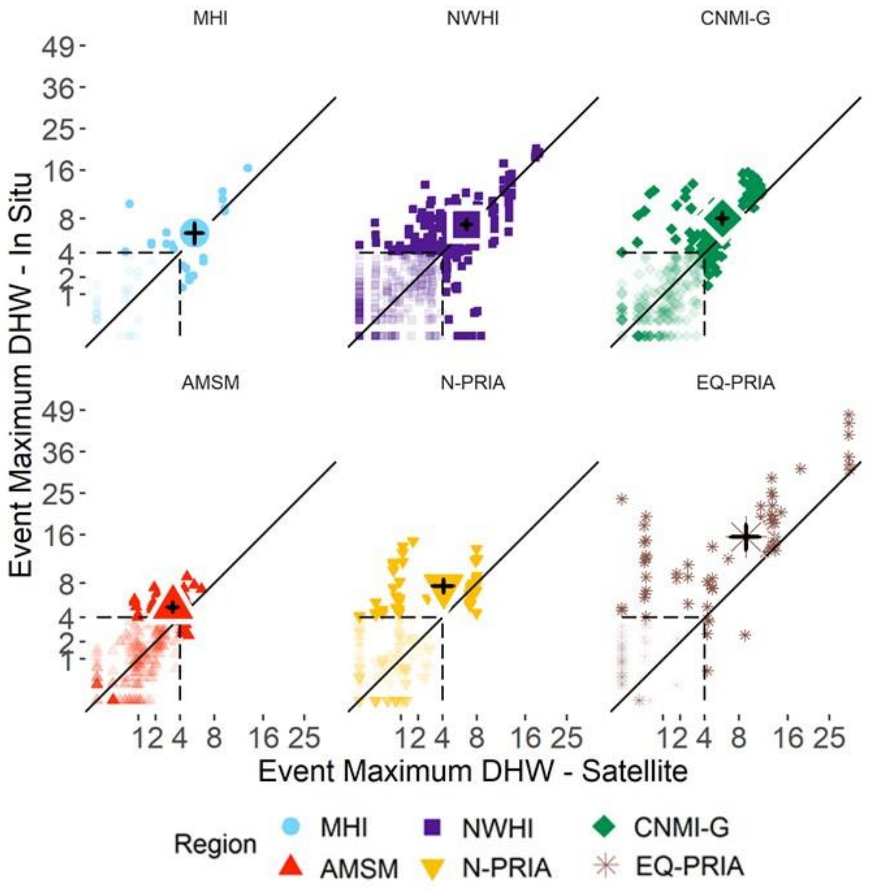

Fig. S5: Surface (satellite) and subsurface (*in situ*) heat stress events for six regions across the western and central Pacific. (n=501 major events total, MHI=36, NWHI=210, CNMI-G=100, AMSM=40, N-PRIA=47, E-PRIA=68). Major events have a maximum severity of 4°C-weeks or greater by either metric and are shown in solid colors. Minor DHW events (<4°C-weeks by both metrics) are shown in pale colors for reference. The regions are identified with different symbols and colors as defined in the legend; region-mean DHW (only major events) is shown by a larger symbol with black whiskers indicating standard error. For comparison, Figure 6a shows the basin-wide analyses.

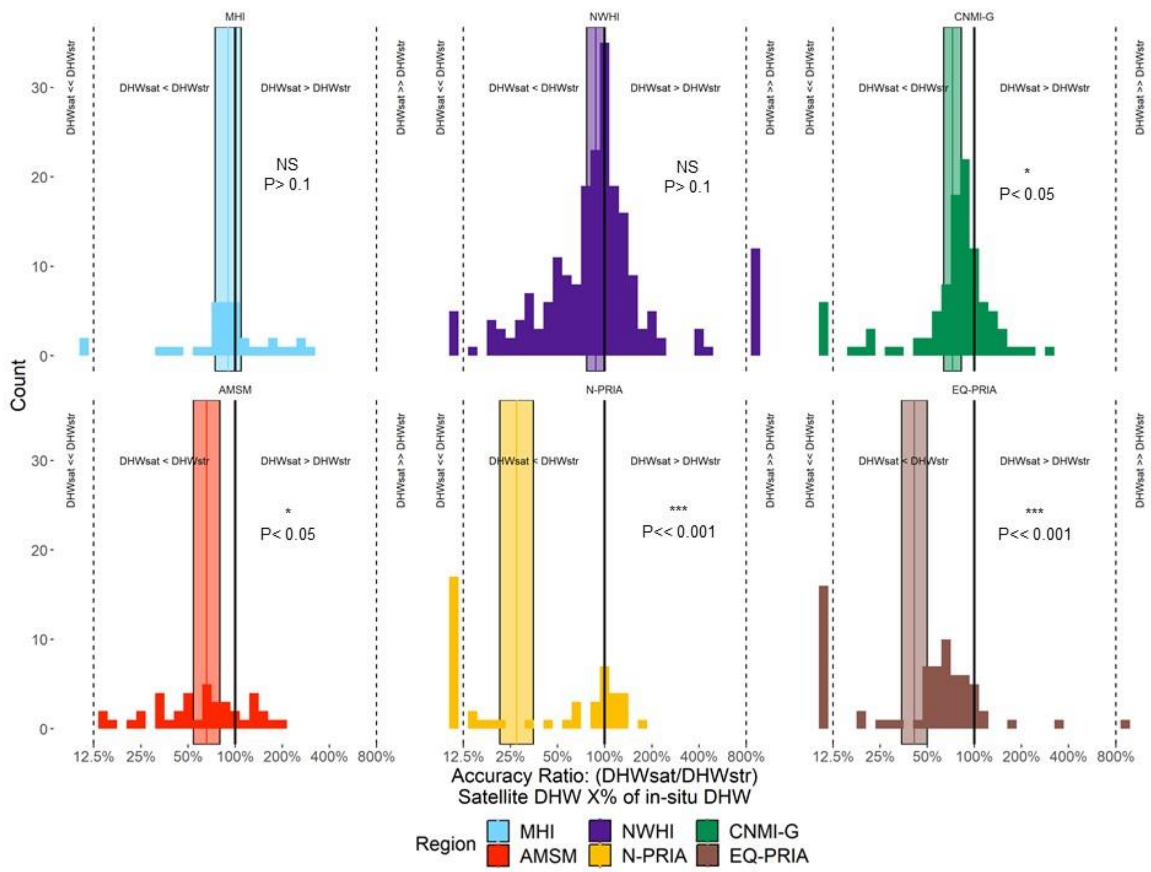

Fig. S6: Distribution of bias between subsurface (STR) and surface (SST) DHW for all six regions across the western and central Pacific, expressed as the Accuracy Ratio of the two metrics, defined as (DHW_{SST}/DHW_{STR}), plotted on a log_2 transformed axis (n=501 major events total, MHI=36, NWHI=210, CNMI-G=100, AMSM=40, N-PRIA=47, E-PRIA=68). Bias left and right of vertical black line centered at zero represents SST under- and over-estimation, respectively. Statistical significance from mixed model results for the bias between STR vs SST at each of the six regions are shown: NS = not statistically significant with p>0.05. ** = statistically significant with p<0.01, and *** = statistically significant with p<<0.001. The regions are identified with different symbols and colors as defined in the legend. The pale colored vertical bar in each panel highlights the mean mixed model estimate of bias with standard error. For comparison, Figure 6b shows the basin-wide analyses.

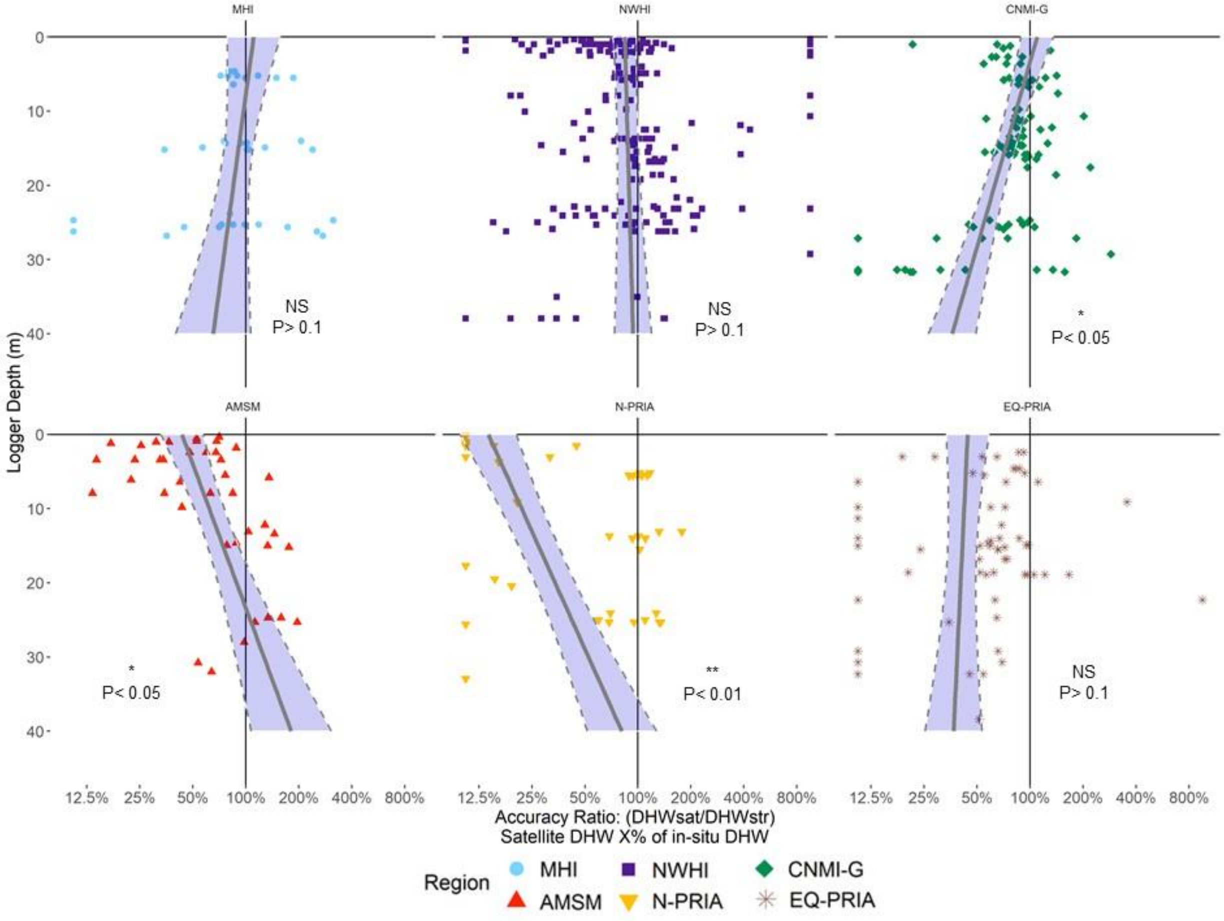

Fig. S7: Association of bias between *in situ* (STR) and satellite (SST) estimates of DHW with depth. Bias is expressed as the Accuracy Ratio of the two metrics, defined as (DHW_{SST}/DHW_{STR}), plotted on a $\log_2$ transformed axis, for six regions across the western and central Pacific (n=501 major events total, MHI=36, NWHI=210, CNMI-G=100, AMSM=40, N-PRIA=47, E-PRIA=68). Statistical significances for the bias between DHW estimates and depths at each of the six regions are shown: NS = not statistically significant with p>0.05. * = statistically significant with p<0.05, and ** = statistically significant with p<0.01. The regions are identified with different symbols and colors as defined in the legend. For comparison, Figure 6c shows the basin-wide analyses.

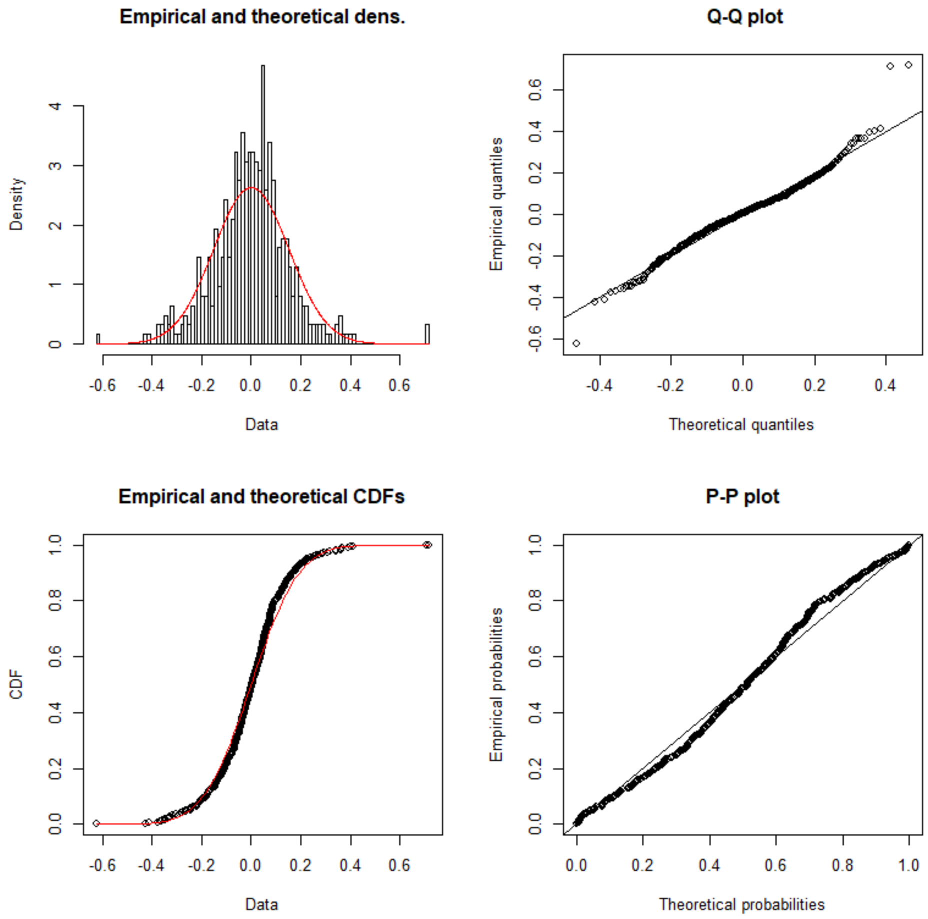

Fig. S8: Summer SST Bias Model Performance Metrics. Statistical performance metrics from linear mixed model of SST bias during the climatologically warmest month (aka “summer”). Statistics underlying data presented in Fig. 2A.

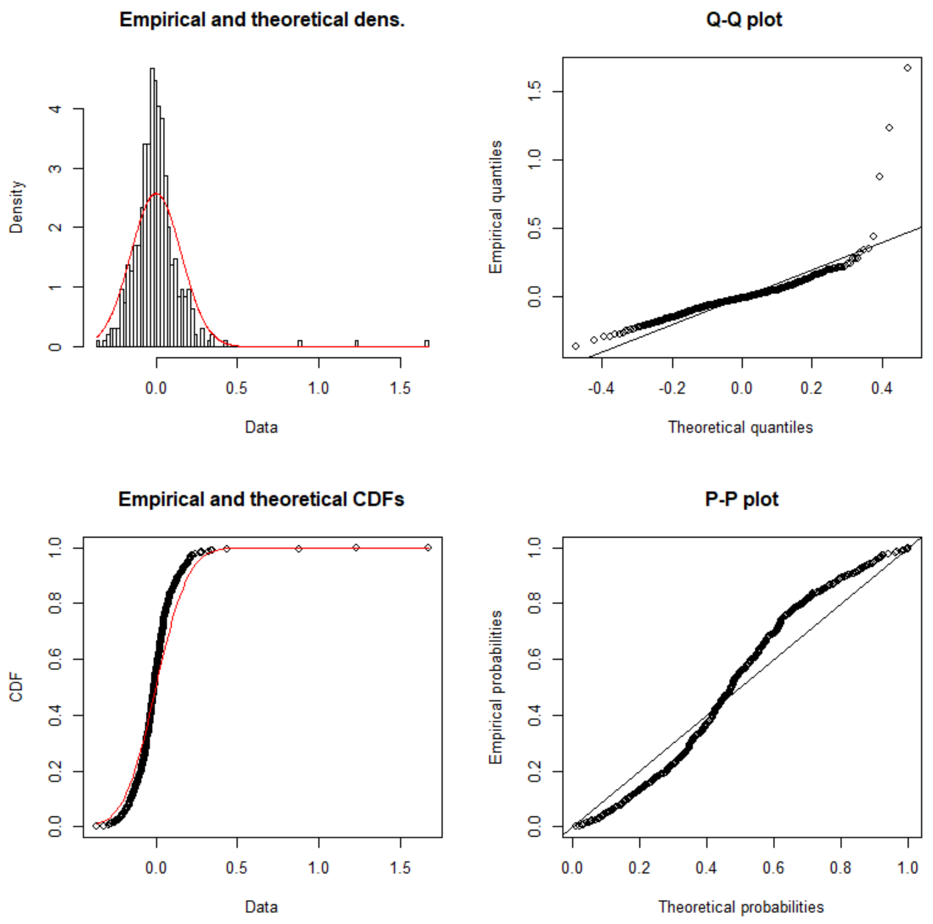

Fig. S9: Winter SST Bias Model Performance Metrics. Statistical performance metrics from linear mixed model of SST bias during the month 6 months offset from the climatologically warmest months (aka “winter). Statistics underlying data presented in Fig. 2B.

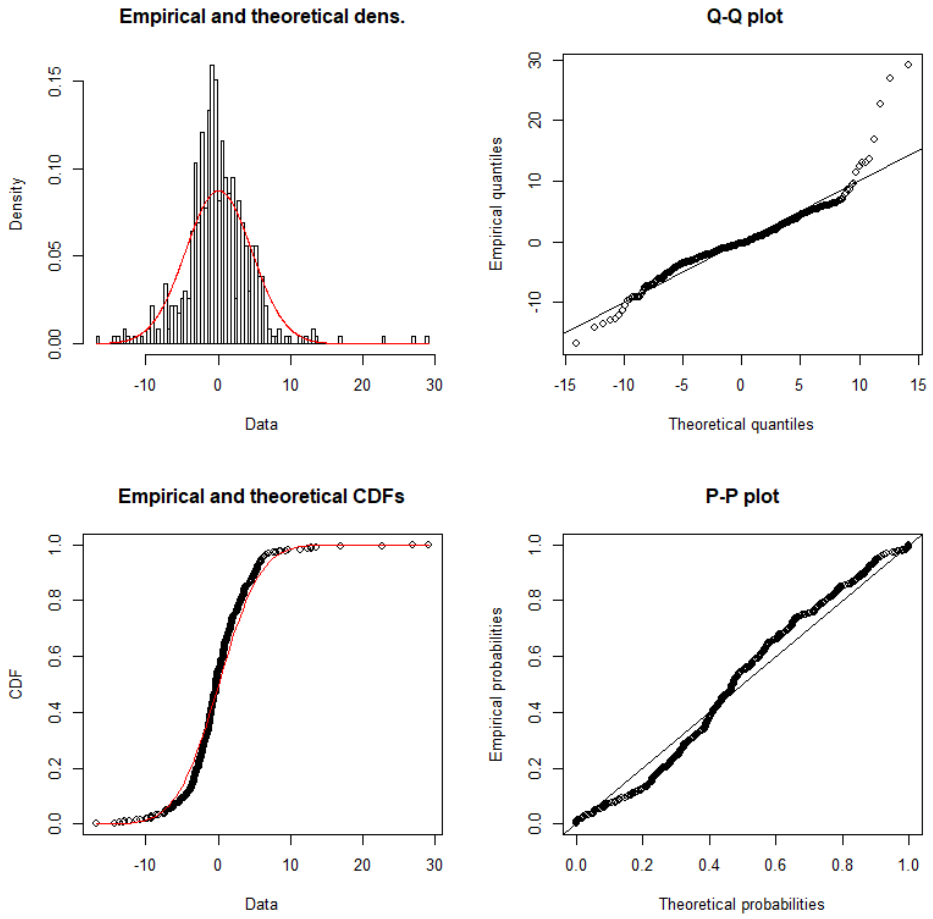

Fig. S10: Maximum DHW vs Depth Model Performance Metrics. Statistical performance metrics from linear mixed model of relationship between Maximum DHW and depth during major warming events. Statistics underlying data presented in Fig. 5.

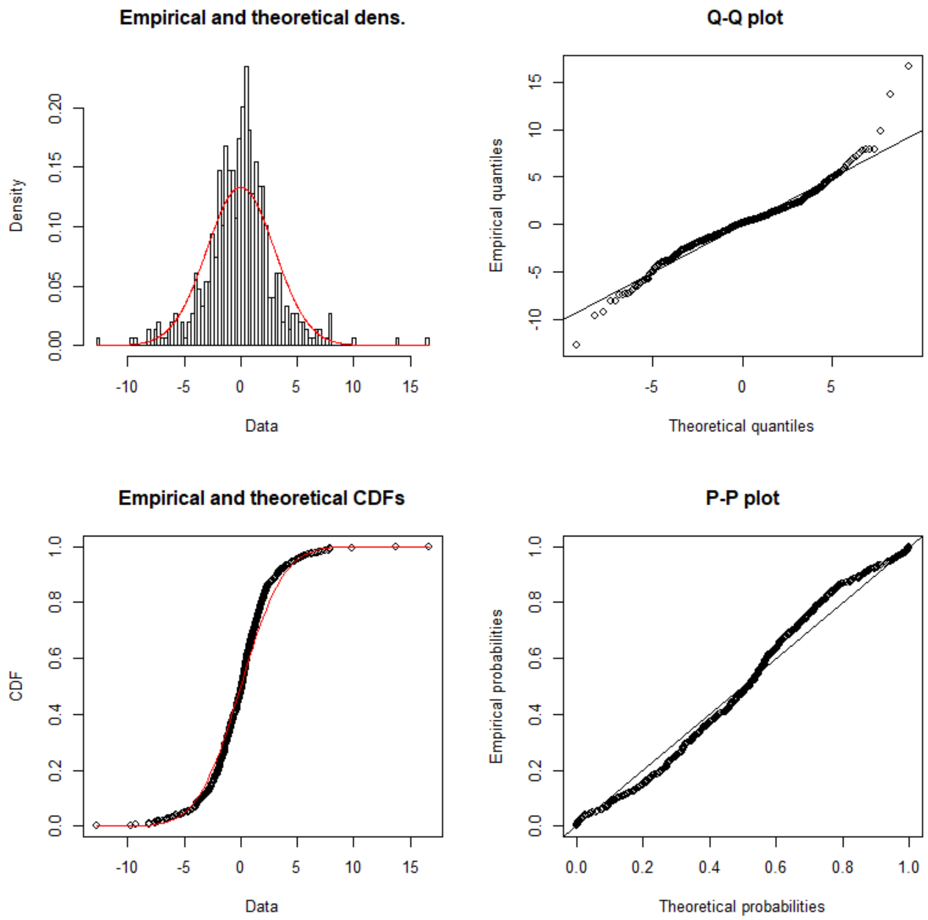

Fig. S11: DHW_{STR} - DHW_{SST} Skew Model Performance Metrics. Statistical performance metrics from linear mixed model of skew between DHW_{STR} and DHW_{SST} during major warming events. Statistics underlying data presented in Fig. 6A.

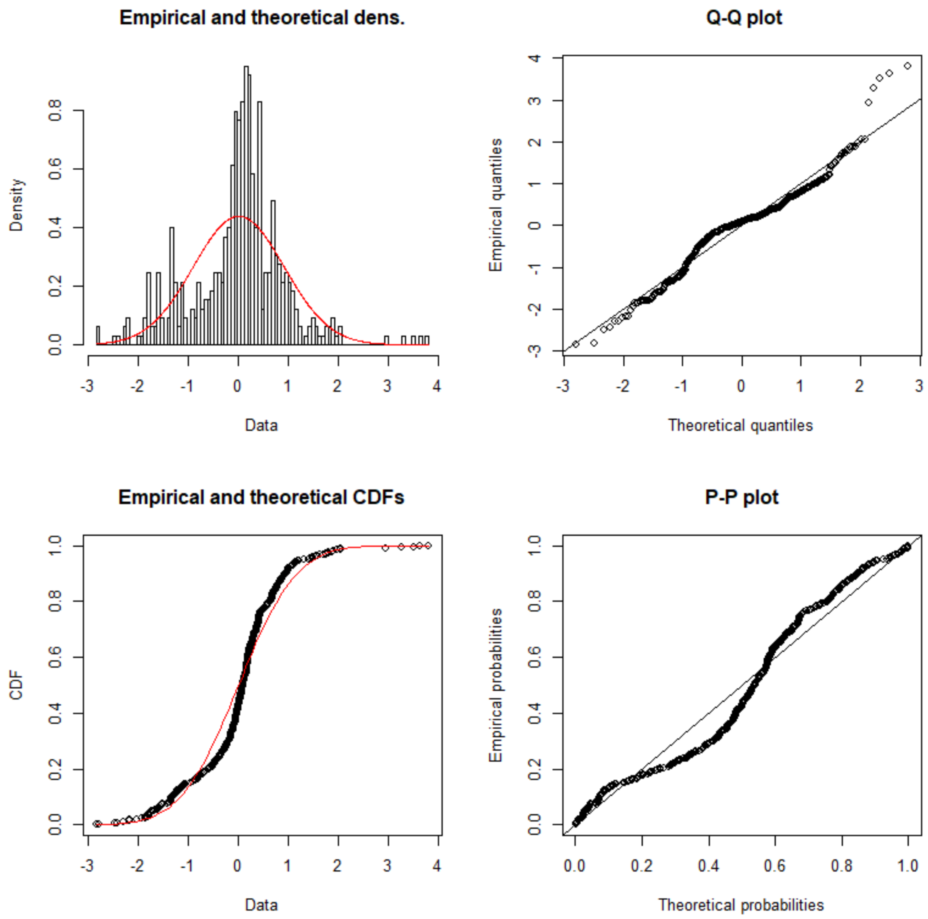

Fig. S12: DHW Accuracy Ratio Model Performance Metrics. Statistical performance metrics from linear mixed model of skew in the DHW Accuracy Ratio during major warming events. Statistics underlying data presented in Fig. 6B.

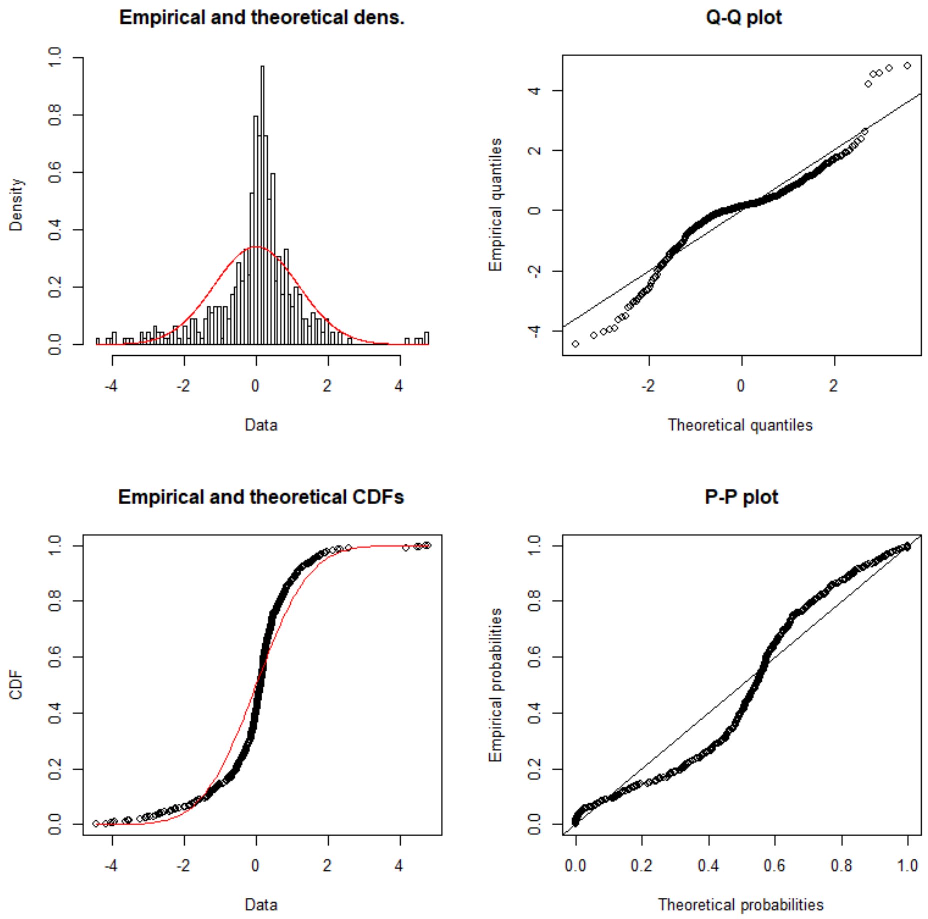

Fig. S13: DHW Accuracy Ratio vs Depth Model Performance Metrics. Statistical performance metrics from linear mixed model of relationship between skew in the DHW Accuracy Ratio and depth during major warming events. Statistics underlying data presented in Fig. 6C.

Table S1: Summer SST Bias Model Fit Summary. Statistical summary tables from linear mixed model of SST bias during the climatologically warmest month (aka “summer”). Statistics underlying data presented in Fig. 2A.

SST Bias: Climatologically Warmest Month

```
Family: gaussian  ( identity )
Formula:          exp_SST_Bias_MM_mn ~ Depth + (1 | Region6P/Island)
Data: Site_Level_Data

     AIC      BIC   logLik deviance df.resid
  -304.6   -284.0    157.3   -314.6      452

Random effects:

Conditional model:
 Groups          Name        Variance  Std.Dev.
 Island:Region6P (Intercept) 1.430e-02 1.196e-01
 Region6P        (Intercept) 2.803e-11 5.294e-06
 Residual                    2.489e-02 1.578e-01
Number of obs: 457, groups:  Island:Region6P, 49; Region6P, 6

Dispersion estimate for gaussian family (sigma^2): 0.0249

Conditional model:
             Estimate Std. Error z value Pr(>|z|)
(Intercept)  0.9882733  0.0233629   42.30   <2e-16 ***
Depth       -0.0110719  0.0008895  -12.45   <2e-16 ***
---
Signif. codes:  0 ‘***’ 0.001 ‘**’ 0.01 ‘*’ 0.05 ‘.’ 0.1 ‘ ’ 1
```

Table S2: Winter SST Bias Model Fit Summary. Statistical summary tables from linear mixed model of SST bias during the month 6 months offset from the climatologically warmest months (aka “winter). Statistics underlying data presented in Fig. 2B.

```
Family: gaussian  ( identity )
Formula:          exp_SST_Bias_6offMM_mn ~ Depth + (1 | Region6P/Island)
Data: Site_Level_Data

     AIC      BIC   logLik deviance df.resid
  -328.0   -307.3    169.0   -338.0      452

Random effects:

Conditional model:
 Groups          Name        Variance Std.Dev.
 Island:Region6P (Intercept) 0.003832 0.06190
 Region6P        (Intercept) 0.002583 0.05082
 Residual                    0.025357 0.15924
Number of obs: 457, groups:  Island:Region6P, 49; Region6P, 6

Dispersion estimate for gaussian family (sigma^2): 0.0254

Conditional model:
             Estimate Std. Error z value Pr(>|z|)
(Intercept)  0.9686587  0.0273913   35.36  < 2e-16 ***
Depth       -0.0025277  0.0008841   -2.86  0.00425 **
---
Signif. codes:  0 ‘***’ 0.001 ‘**’ 0.01 ‘*’ 0.05 ‘.’ 0.1 ‘ ’ 1
```

Table S3: Maximum DHW vs Depth Model Fit Summary. Statistical summary tables from linear mixed model of relationship between Maximum DHW and depth during major warming events. Statistics underlying data presented in Fig. 5.

```
Family: nbinom2  ( log )
Formula:          MaxDHW_STR_rnd ~ Depth + (1 | Region6P/Island/SiteID)
Zero inflation:                  ~1
Data: MajorEvents

     AIC      BIC   logLik deviance df.resid
  2792.2   2821.7  -1389.1   2778.2      494

Random effects:

Conditional model:
 Groups                   Name        Variance  Std.Dev.
 SiteID:(Island:Region6P) (Intercept) 8.284e-10 2.878e-05
 Island:Region6P          (Intercept) 8.064e-02 2.840e-01
 Region6P                 (Intercept) 8.890e-02 2.982e-01
Number of obs: 501, groups:  SiteID:(Island:Region6P), 257; Island:Region6P, 40; Region6P, 6

Overdispersion parameter for nbinom2 family ():  9.5

Conditional model:
             Estimate Std. Error z value Pr(>|z|)
(Intercept)  2.008802   0.140733  14.274   <2e-16 ***
Depth       -0.001585   0.002326  -0.682    0.496
---
Signif. codes:  0 ‘***’ 0.001 ‘**’ 0.01 ‘*’ 0.05 ‘.’ 0.1 ‘ ’ 1

Zero-inflation model:
            Estimate Std. Error z value Pr(>|z|)
(Intercept)  -3.9875     0.3894  -10.24   <2e-16 ***
---
Signif. codes:  0 ‘***’ 0.001 ‘**’ 0.01 ‘*’ 0.05 ‘.’ 0.1 ‘ ’ 1
```

Table S4: DHW_{STR} - DHW_{SST} Skew Model Fit Summary. Statistical summary tables from linear mixed model of skew between DHW_{STR} and DHW_{SST} during major warming events. Statistics underlying data presented in Fig. 6A.

```
Family: gaussian (identity)
Formula:          MaxDHW.depth.MMadj ~ MaxDHW.sst + (1 | Region6P/Island/SiteID)
Data: MajorEvents

     AIC      BIC   logLik deviance df.resid
  2672.9   2698.2  -1330.5   2660.9      495

Random effects:

Conditional model:
 Groups                   Name        Variance Std.Dev.
 SiteID:(Island:Region6P) (Intercept)  0.8447  0.9191
 Island:Region6P          (Intercept)  1.8752  1.3694
 Region6P                 (Intercept)  4.2050  2.0506
 Residual                             10.0229  3.1659
Number of obs: 501, groups:  SiteID:(Island:Region6P), 257; Island:Region6P, 40; Region6P, 6

Dispersion estimate for gaussian family (sigma^2):   10

Conditional model:
            Estimate Std. Error z value Pr(>|z|)
(Intercept)  3.50921    0.92451   3.796 0.000147 ***
MaxDHW.sst   0.79401    0.03541  22.422  < 2e-16 ***
---
Signif. codes:  0 ‘***’ 0.001 ‘**’ 0.01 ‘*’ 0.05 ‘.’ 0.1 ‘ ’ 1
```

Table S5: DHW Accuracy Ratio Model Fit Summary. Statistical summary tables from linear mixed model of skew in the DHW Accuracy Ratio during major warming events. Statistics underlying data presented in Fig. 6B.

**** 0/X, X/0 set to outlier value (log2(-3),log2(3)) ****

```
Family: gaussian  ( identity )
Formula:          LRBias_PLOT ~ 1 + (1 | Region6P/Island/SiteID)
Zero inflation:              ~1
Data: MajorEvents[which(!is.infinite(MajorEvents$LRBias)), ]

     AIC      BIC   logLik deviance df.resid
  1492.6   1517.7   -740.3   1480.6      480

Random effects:

Conditional model:
 Groups                   Name        Variance Std.Dev.
 SiteID:(Island:Region6P) (Intercept) 0.19892  0.4460
 Island:Region6P          (Intercept) 0.09396  0.3065
 Region6P                 (Intercept) 0.21874  0.4677
 Residual                             0.98431  0.9921
Number of obs: 486, groups:  SiteID:(Island:Region6P), 253; Island:Region6P, 40; Region6P, 6

Dispersion estimate for gaussian family (sigma^2): 0.984

Conditional model:
            Estimate Std. Error z value Pr(>|z|)
(Intercept)  -0.6358     0.2119  -3.001  0.00269 **
---
Signif. codes:  0 '***' 0.001 '**' 0.01 '*' 0.05 '.' 0.1 ' ' 1

Zero-inflation model:
            Estimate Std. Error z value Pr(>|z|)
(Intercept)   -22.71    3879.93  -0.006    0.995
```

Table S6: DHW Accuracy Ratio vs Depth Model Fit Summary. Statistical summary tables from linear mixed model of relationship between skew in the DHW Accuracy Ratio and depth during major warming events. Statistics underlying data presented in Fig. 6C.

Family: gaussian (identity)
Formula: LRBias_PLOT ~ Depth + (1 | Region6P/Island/SiteID)
Data: MajorEvents

AIC	BIC	logLik	deviance	df.resid
1629.6	1654.9	-808.8	1617.6	495

Random effects:

Conditional model:

Groups	Name	Variance	Std.Dev.
SiteID:(Island:Region6P)	(Intercept)	0.1555	0.3943
Island:Region6P	(Intercept)	0.1257	0.3545
Region6P	(Intercept)	0.2857	0.5345
Residual		1.2425	1.1147

Number of obs: 501, groups: SiteID:(Island:Region6P), 257; Island:Region6P, 40; Region6P, 6

Dispersion estimate for gaussian family (sigma^2): 1.24

Conditional model:

	Estimate	Std. Error	z value	Pr(>|z|)	
(Intercept)	-0.701492	0.254411	-2.757	0.00583	**
Depth	0.002816	0.006108	0.461	0.64479	

Signif. codes: 0 ‘***’ 0.001 ‘**’ 0.01 ‘*’ 0.05 ‘.’ 0.1 ‘ ’ 1
